# Supplementary material for: Ultra-low threshold lasing through phase front engineering via a metallic circular aperture
Source: Nat Commun. 2022 Jan 11;13:230. doi: 10.1038/s41467-021-27927-9 (PMC8752788; doi:10.1038/s41467-021-27927-9)
Supplement: Supplementary file 1 — Supplementary Information [file 41467_2021_27927_MOESM1_ESM.pdf]

Supplemental Material: Ultra-low threshold lasing through phase front engineering via a metallic circular aperture

Zhixin Wang[1,*], **Filippos Kapsalidis**[1], **Ruijun Wang**[1], **Mattias Beck**[1], **and Jérôme Faist**[1,*]

[1]ETH Zürich, Institute of Quantum Electronics, Auguste-Piccard-Hof 1, Zürich 8093, Switzerland
[*]corresponding author: Zhixin Wang (zhixwang@phys.ethz.ch); Jérôme Faist (jfaist@ethz.ch)

A Additional data and explanation of the reflectivity enhancement

A.1 Beam focusing by the metallic aperture

As mentioned in the main text, the optimized metallic aperture behaves effectively as a lens which refocuses the reflected beam, decreases the diffraction loss, and enhances the modal reflectivity. This mechanism is schematically illustrated in Fig. S1. In the case of a flat metallic coating, the beam diverges within the dielectric layer, as shown in Fig. S1(a), and the diffraction loss arises. In the case where an optimized aperture in opened in the metal film of the coating, the divergence of the reflected beam is reduced due to the phase correction of the electromagnetic dipole resonance as mentioned in the main text . This is physically equivalent as focusing the back-reflected beam, where the metallic aperture behaves as a lens, in an effective reminiscence of the Arago spot, as illustrated in Fig. S1(b). In the following paragraphs, this focusing effect will be clearly explained in details.

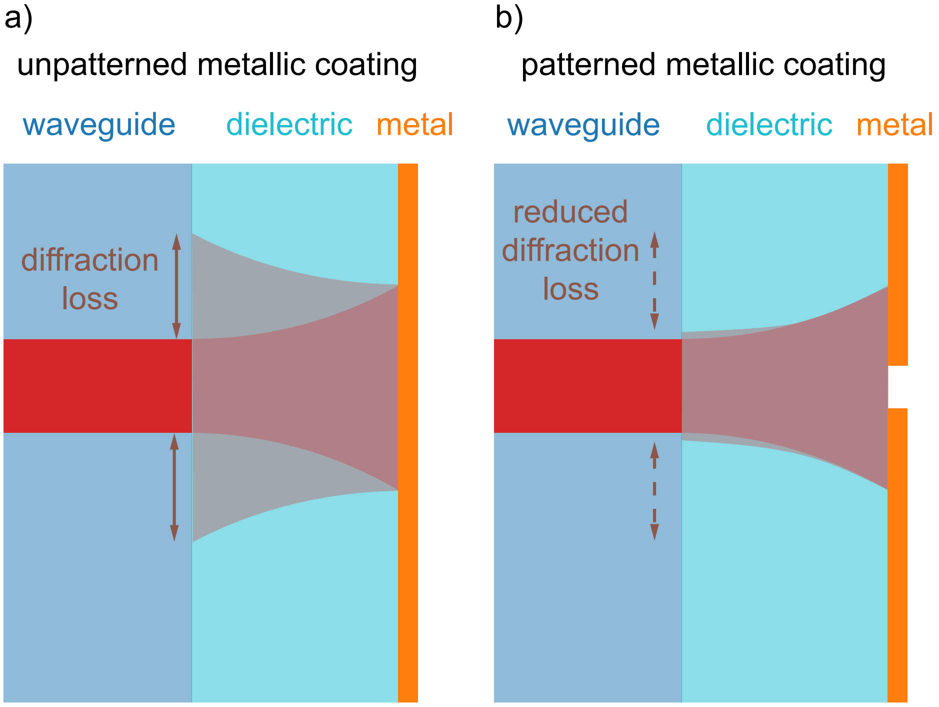

Figure S1. **Effective focusing.** Schematic drawing of the mechanism that the optimized metallic aperture behaves effectively as a lens, focuses the diverging beam and reduces the diffraction loss. The red rectangle in the left region of both plots represent the active region waveguide inside the laser, surrounded by the low-refractive-index cladding (dark blue). The light blue region in both plots represents the insulating layer in the metallic coating. The beam divergence inside the insulating layer is schematically depicted by the semi-transparent red geometry. The orange region on the right side of both plots represent the metal films.

Equivalent approach to resolve the reflected beam shape

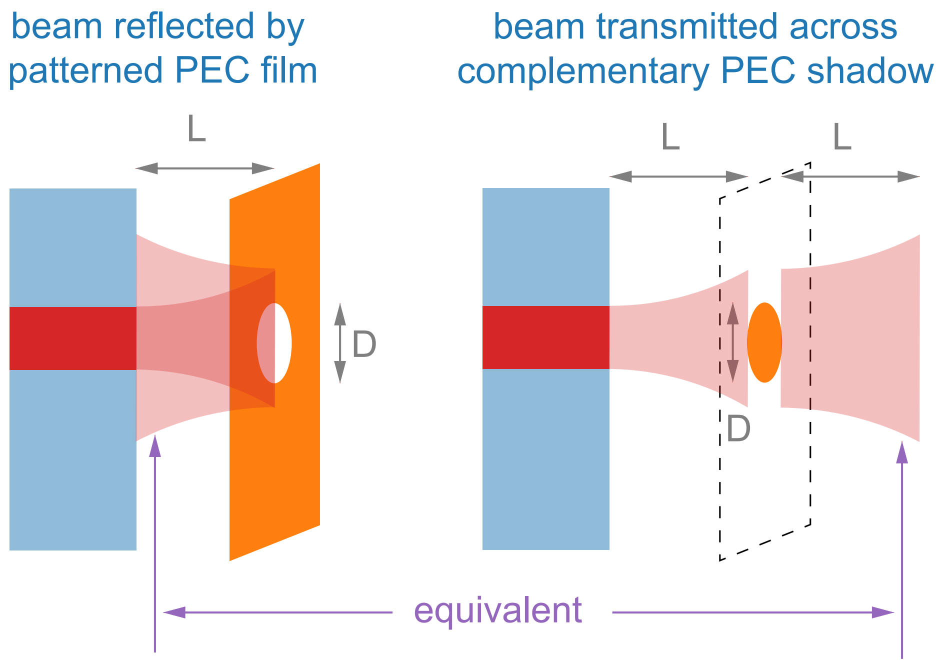

Figure S2. Unfolding the reflected field. Schematic drawing which shows that the beam reflected by a patterned PEC (perfect electric conductor) film is equivalent to the beam transmitted across a PEC shadow with the complementary pattern. The active region waveguide (red) inside the laser is surrounded by the low-index cladding (blue). The semi-transparent red geometry represents the beam emitted out of the waveguide, reflected by the metal film (orange in the left plot) or transmitted across the metal shadow (orange in the right plot). L represents the distance between the waveguide facet and the metal film (left plot) or the metal shadow (right plot), and D is the diameter of the metal aperture (left plot) or the metal shadow (right plot).

The focusing effect by the metallic aperture can be proved by comparing the shapes of the reflected beam. However, it is technically difficult to directly retrieve purely the reflected beam with high precision and accuracy from the 3D COMSOL model, because it is mixed with the incident beam inside the dielectric layer, and the situation becomes more complicated especially at the waveguide interface. Instead, the reflected beam shape at the interface can be resolved using an equivalent approach, as illustrated in Fig. S2.

In the ideal case where we assume the metal film as perfect electric conductor (PEC) and neglect the reflection within the aperture (the reflectivity between Al_2O_3 and air is 5%), the beam reflected by a patterned PEC film is equivalent to the beam transmitted across a PEC shadow with the complementary shape as the milled pattern, because of the same boundary conditions for the diffraction. In the left plot of Fig. S2, the dielectric material separating the semiconductor waveguide and the metal film is Al_2O_3. In the right plot of Fig. S2, the medium before and after the PEC shadow are both Al_2O_3. The distance between the waveguide interface and the PEC film (shadow) equals to L in both plots. The pattern in the PEC film (left) and the PEC shadow (right) are both in circular shape with the same diameter D and are both aligned to the center of the waveguide. With the mentioned assumptions, the shape of the reflected beam arriving at the waveguide interface (left figure) is the same as the the shape of the transmitted beam with a propagation distance L after the PEC shadow (right figure). This plane is also referred to as the *target plane* in the following discussion. Different to the reflected beam which is mixed with the incident light, the transmitted beam is separated from that. Because the beam shapes at both target planes are identical, this equivalent approach allows us to unwrap the reflected beam and investigate its shape with high precision.

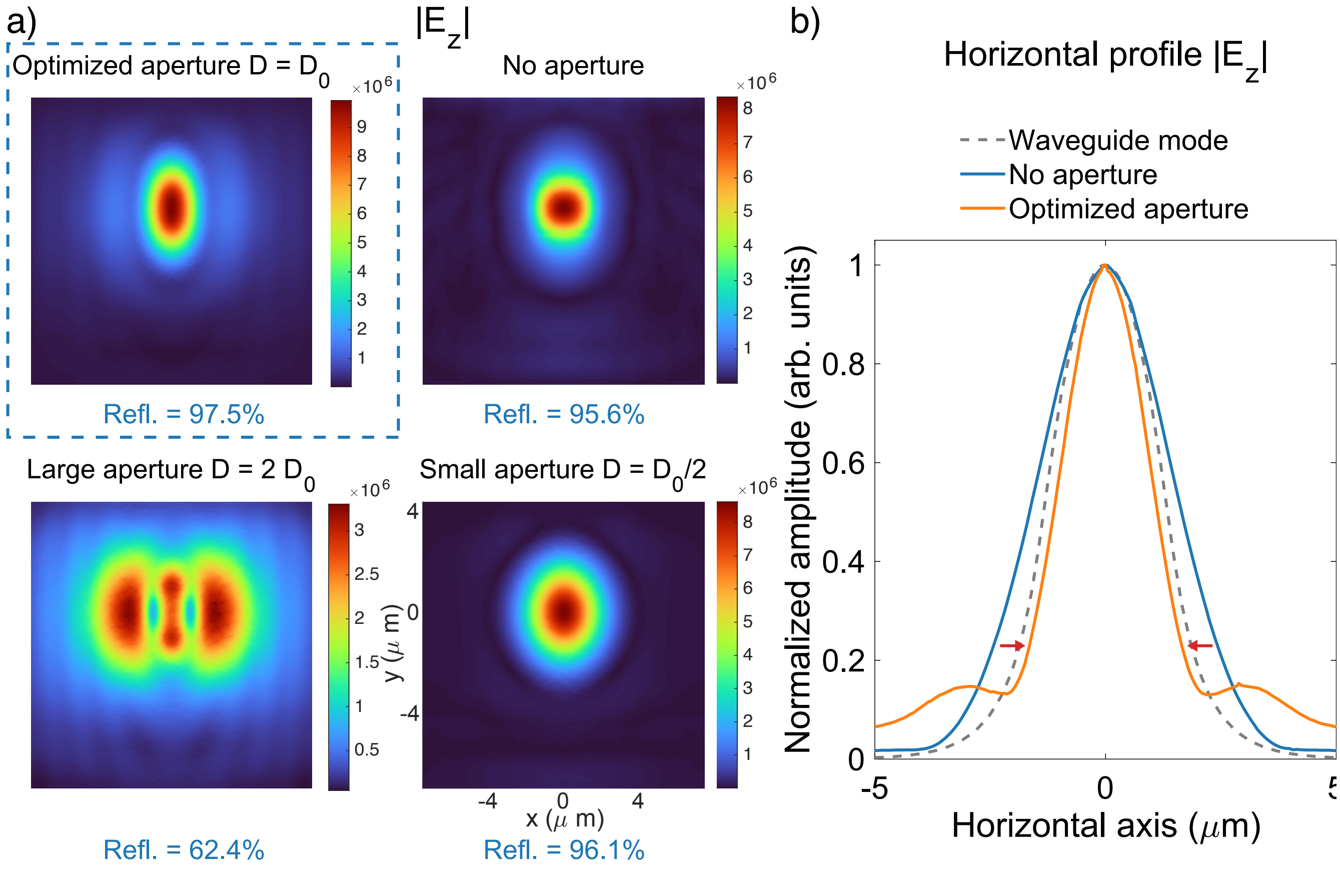

Figure S3. **Beam narrowing with an optimized aperture.** (a) Beam shape (absolute value of the vertical electric field component $|\mathbf{E}_z|$) at the target plane with four different aperture diameters D: optimized aperture diameter $D = D_0 = 950$ nm; no aperture; large aperture $D = 2D_0$; small aperture $D = D_0/2$. The beam shape is simulated with 3D COMSOL where the dielectric layer thickness $L = 700$ nm. The modal reflectivity is labeled in each case. The coordinate axis scales in four plots are all the same. (b) Horizontal profiles ($|\mathbf{E}_z|$ on the axis of $y = 0$) of: the waveguide mode, the reflected beam with no aperture, and the reflected beam with an optimized aperture.

Beam shape dependence on aperture diameter D

Using this approach, we investigated the dependence of the beam shape at the target plane on D, L and during the propagation. Figure S3(a) shows the beam shapes with L = 700 nm ($\approx \lambda/4$) and four different aperture conditions: optimized aperture diameter $D = D_0 = 950$ nm; large aperture $D = 2D_0$; small aperture $D = D_0/2$ and no aperture. Shown in these plots are the absolute values of the vertical electric field ($|\mathbf{E}_z|$), because the waveguide modes are TM-polarized.

Compared to the beam shape without any aperture [top right plot in Fig. S3(a)], the beam with an optimized aperture is narrowed in the horizontal direction [top left plot in Fig. S3(a), marked by the blue dashed box]. Figure S3(b) shows the narrowing of the beam in a more clear way, where the horizontal profiles on the $y = 0$ axes are plotted for three cases: the waveguide mode (gray dashed line), without any aperture (the blue line) and with the optimized aperture (the orange line). As indicate by the red arrows, the metallic aperture narrows the beam width and leads to a better coupling with the waveguide mode.

The feature that the focusing occurs mainly in the horizontal direction agrees with the polarization properties of the system. As shown in Fig. 3 of the main text, the electric field is mainly vertically polarized, and the continuous boundary conditions of the tangential electric field push the light away from left and right poles of the aperture. Near these two points, the electric field is much weaker than the case without any aperture. With the optimized parameters, such a change results in a narrowing of the beam as shown in Fig. S3(b). This focusing effect disappears if the aperture is too large or too small, as shown in the bottom two plots of Fig. S3(a).

Beam shapes at different planes

The focusing effect of the metallic aperture can be again confirmed by comparing the beam profiles in different planes during the propagation (equivalently, reflection), as shown in Fig. S4. Here the dielectric layer thickness $L = 700$ nm and aperture diameter D = 950 nm. The left figures are the horizontal profiles [defined in the same way as Fig. S3(b)] at four different planes: the waveguide mode, half-way to the aperture (350 nm away), at the aperture, and at the target plane (arriving at the waveguide interface for the reflected beam).

A comparison between the top two plots in Fig. S4 indicates that the beam gets broader while propagating in the dielectric layer as the waveguide confinement is lifted. The diverging beam is refocused by the metallic aperture to be even narrower than the initial waveguide mode, as shown by the bottom plot of the figure.

Beam shape dependence on the dielectric layer thickness L

We further analyzed the dependence of the beam shape at the target plane on the dielectric layer thickness L, as plotted in Fig. S5. The figure marked by the dashed box is the beam shape with the optimized parameters, which is the same as the plot of Fig. S3(a) marked in the same way. Figure S5 shows that when L is too small, two off-center lobes are clearly observed along the vertical symmetry axis of the beam. For the aperture diameter $D = 950$ nm, the beam shape becomes single-lobed with L near or larger than 700 nm. On the other hand, the beam gets much broader if L is way larger than the 700 nm. Therefore, the aperture diameter must be matched with the dielectric layer thickness in order to achieve the optimal reflectivity, as shown in Fig. S6.

Summary

Figures S3, S4, S5 confirm our explanation that the reflected beam is refocused by an optimized metallic aperture. This effect mainly occurs in the horizontal direction of the beam. Focusing the reflected beam at the waveguide interface enhances the efficiency of the coupling back into the waveguide, reduces the diffraction loss and increases the modal reflectivity. It is physically equivalent to flattening the phase front, which is discussed in the main text of the manuscript.

A.2 Optimized aperture diameters at each insulating layer thickness in Fig. 1(b)

As the complementary data to Fig. 1(b) (dark-blue circled line and red dotted line) in the main text, Fig. S6 shows the selected aperture diameters which maximize the modal reflectivity at each Al_2O_3 layer thickness. The step in the diameter of the

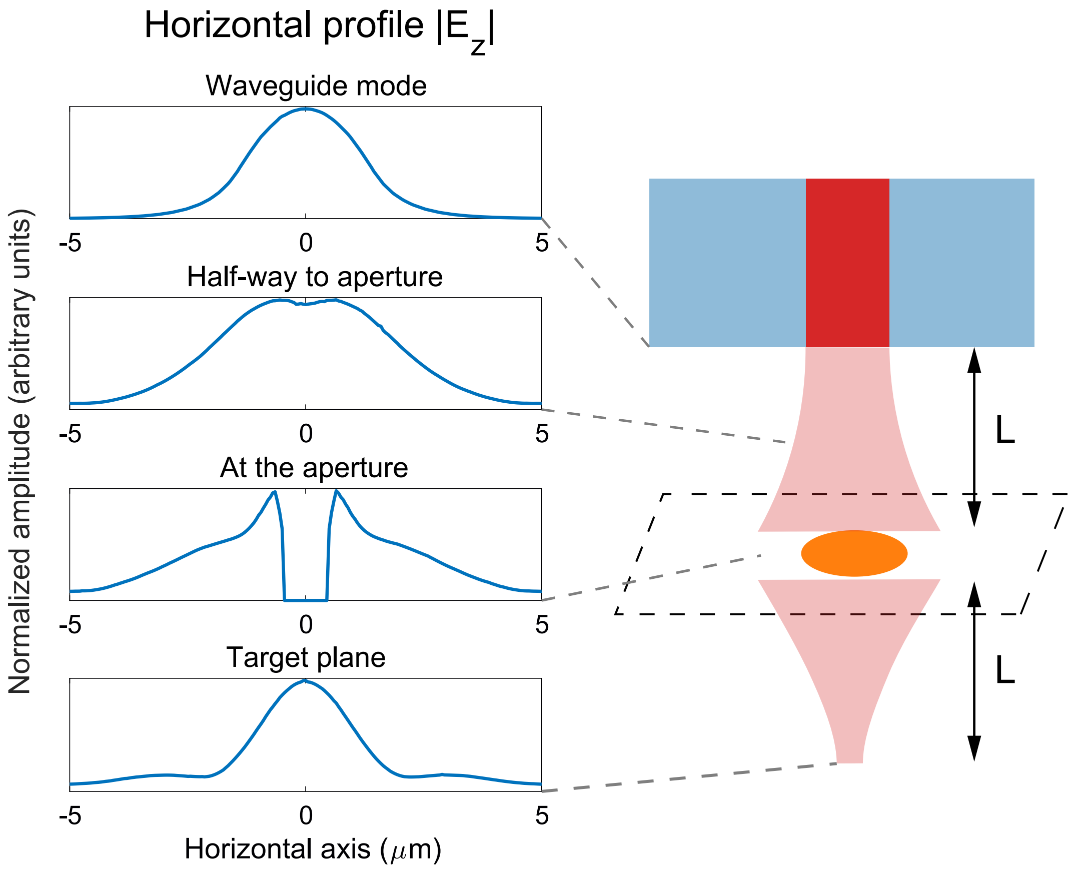

Figure S4. **Beam width during the propagation.** Horizontal beam profiles of the vertical electric field components ($|\mathbf{E}_z|$) at different propagation planes, showing that the optimized metallic aperture behaves as a lens and refocuses the beam. Here the dielectric layer thickness $L = 700$ nm, aperture diameter $D = 950$ nm. The active region waveguide (red) inside the laser is surrounded by the low-index cladding (blue). The semi-transparent red geometry represents the beam emitted out of the waveguide, transmitted across the metal shadow (orange).

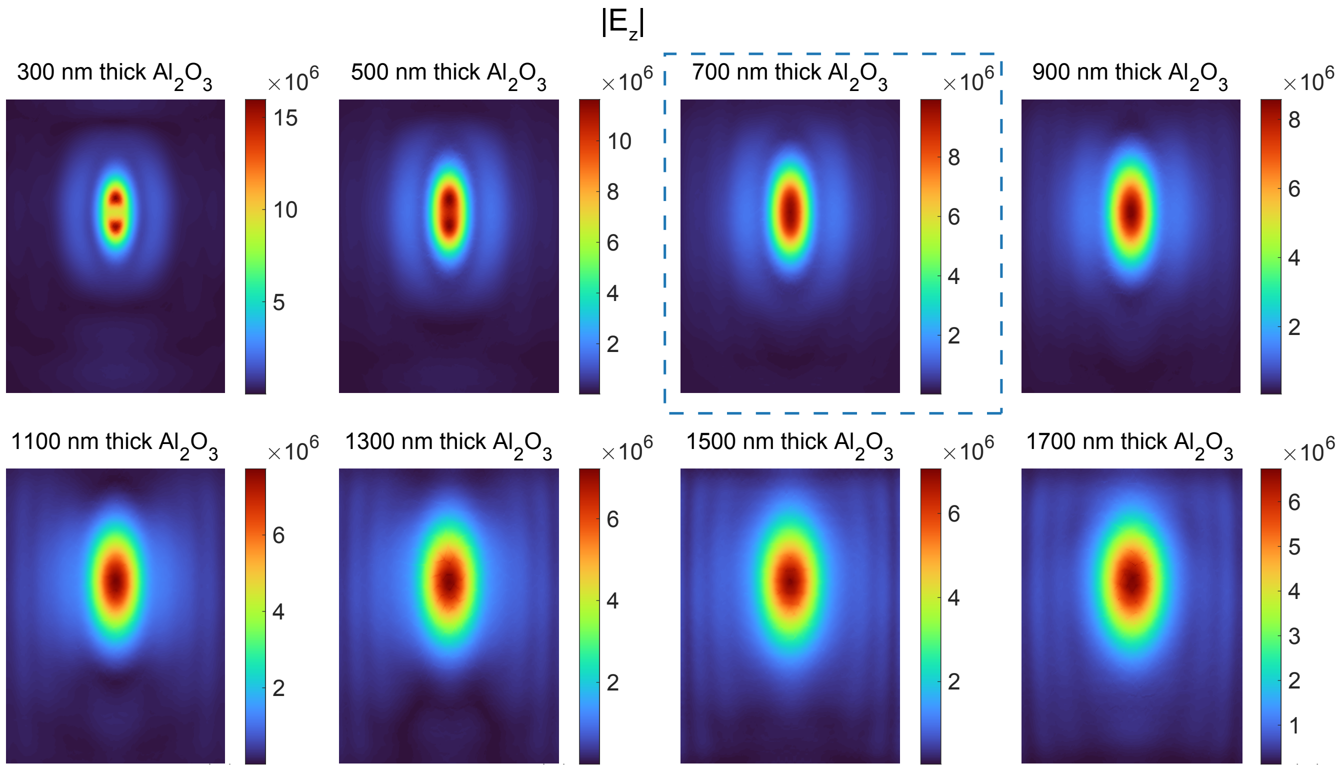

Figure S5. Reflected beam shape with varying dielectric layer thickness. Beam shapes (absolute value of the vertical electric field component $|\mathbf{E}_z|$) at the target plane with different dielectric layer thicknesses. The beam shape is simulated with 3D COMSOL where aperture diameter $D = 950$ nm. The axis of all plots in (a) are the same as Fig. S3(a). The plot in the dashed box shows the beam shape with the optimized parameters.

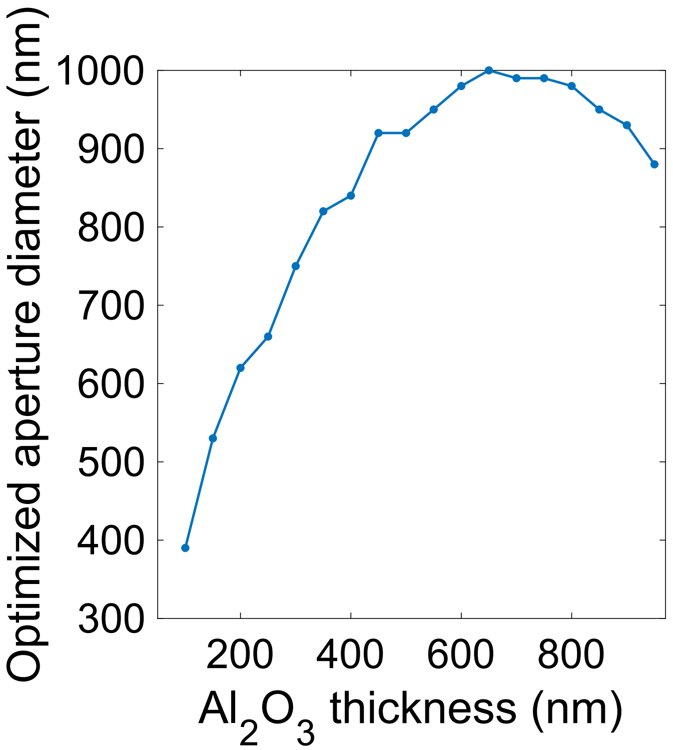

Figure S6. Aperture parameter. Circular aperture diameters with the maximum modal reflectivity for varying Al_2O_3 layer thickness, as the complementary data of Fig. 1(b) in the main text. The step of the parametric scan in the simulation is 10 nm.

parametric scan in the simulation is 10 nm. Discontinuity in the optimized aperture dimensions as a function of the Al_2O_3 thickness results in the discontinuity in the transmission efficiency (red dotted line) in Fig. 1(b).

A.3 Cross-sectional field patterns with different aperture dimensions

As the complementary results to Fig. 1(d) in the main text, Fig. S7 shows the side-view images of $\mathbf{E}_z$ field patterns with 5 different aperture sizes, simulated by COMSOL Multiphysics. It is clearly shown that the curvature of the wavefront changes its sign as the aperture dimension gradually gets larger and the maximum reflectivity is obtained when the wavefront becomes flat (the middle plot). The first, third and fifth plots here are the cases of $d < d_0$, $d \approx d_0$ and $d > d_0$ in Fig. 1(d) of the main text.

B Analysis on the aperture shape

In the main text, we focus on the circular aperture shape because our experimental tool for the FIB milling, the Helios 5 UX, prefers a circular pattern to elliptical shapes. But in principle, the choice of circular shape is not mandatory. We also analyzed other aperture shapes with 3D COMSOL simulation. Because the waveguide shape is horizontally symmetric and quasi-symmetric in the vertical direction, the explored aperture shape is set as ellipses, which are symmetrical along both vertical and horizontal directions.

Figure S8(a) shows the value of the modal reflectivity enhanced by the aperture in the unit of percentage, for varying aperture widths and heights. Figure S8(b) shows the results of the transmissivity in the same simulations. Parameters near the bottom boundaries of the plots represent vertical slit-like aperture shapes and parameters near the left boundaries of the figure represent short but wide aperture shapes. In the blank region of Fig. S8(a), the modal reflectivity is not enhanced compared to the case without the aperture. The black dashed lines show the parameters where the aperture is circular and the red star indicates the parameters we experimentally used in Fig. 2 and Fig. 3 of the main text, where both the aperture height and the width are 950 nm. The difference between the reflectivity with the circular aperture (red star) and the maximum one with the

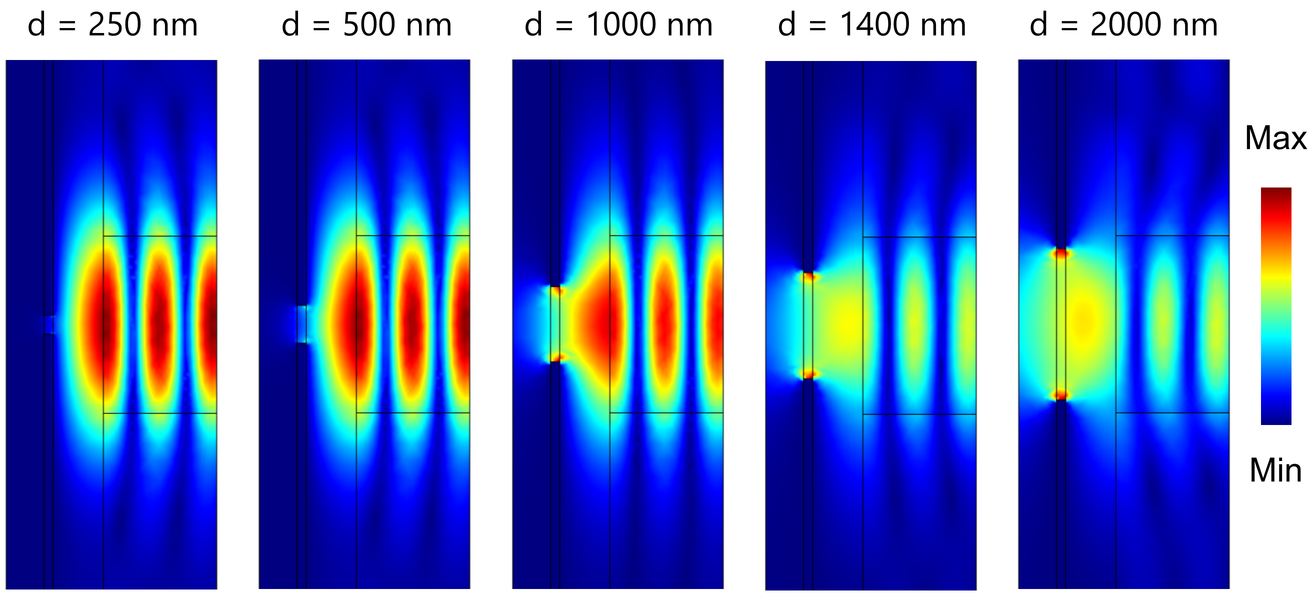

Figure S7. Curvature of the wave-front. Side-view plots of the vertical component of the electric field ($|\mathbf{E}_z|$) in a simulation with 5 aperture sizes, simulated by COMSOL Multiphysics, where d represents the aperture diameter in each simulation. The first, third and fifth plots here are the ones of $d < d_0$, $d \approx d_0$ and $d > d_0$ in Fig. 1(d) of the main text. The colorbar ranges are $[37, 2.4 \times 10^7]$, $[35, 2.5 \times 10^7]$, $[3.8 \times 10^2, 2.8 \times 10^7]$, $[2.9 \times 10^2, 4.1 \times 10^7]$, $[1.4 \times 10^3, 3.9 \times 10^7]$ (arbitrary units), respectively.

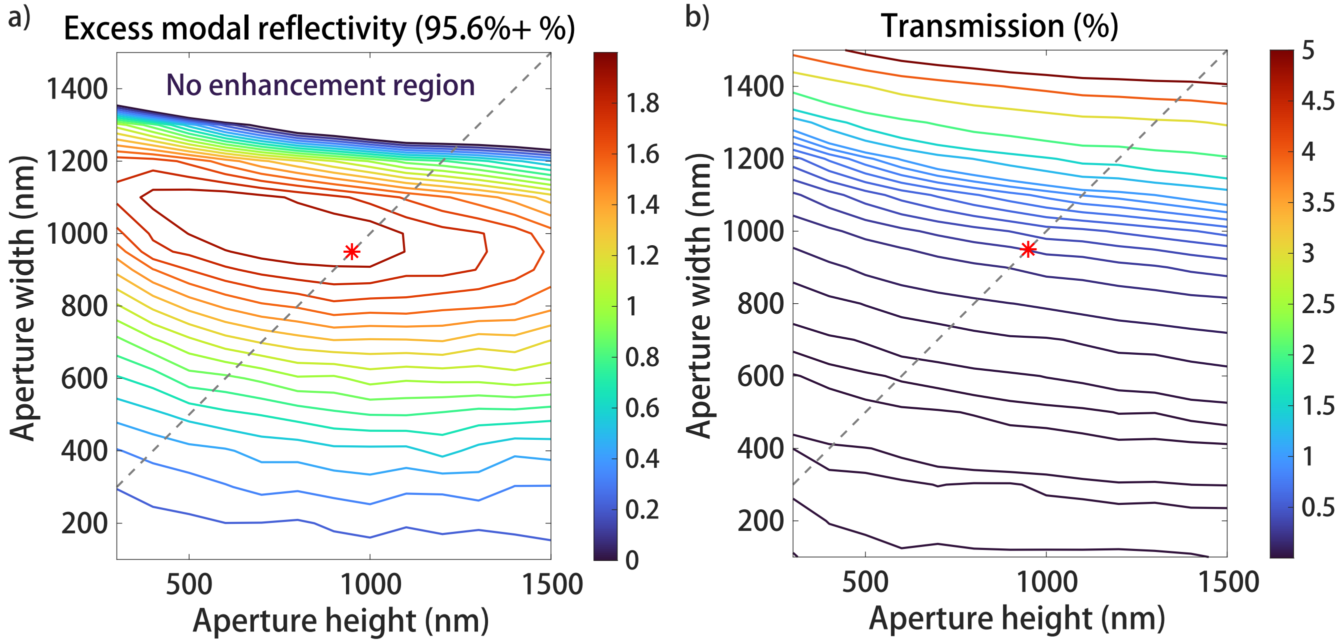

Figure S8. Impact of the aperture shape. (a) The enhanced modal reflectivity for the patterned metallic coating compared to the one without patterning, as a function of the elliptical aperture width and height. The Al_2O_3 layer thickness is 700 nm. The modal reflectivity of an unpatterned metallic coating is 95.6%. The black dashed line shows the parameters where the aperture is circular and the red star shows the parameters chosen in our experiments where both the height and the width is 950 nm. The blank region shows the parameters where the modal reflectivity is not enhanced. (b) Transmissivity of the patterned coating as a function of the elliptical aperture width and height. The simulation settings are the same as (a). The black dashed line shows the parameters where the aperture is circular and the red star shows the parameters chosen in our experiments where both the height and the width is 950 nm.

elliptical aperture (800 nm high, 1000 nm wide) is less than 0.05%. The transmission efficiency of the circular aperture with 950 nm diameter is lower than the one of the elliptical aperture (800 nm high, 1000 nm wide) by 3%. Therefore, the circular aperture is almost as good as the elliptical one but much easier to be manufactured.

Figures S8 (a,b) show that the modal reflectivity and the transmissivity is more sensitive to the width of the aperture than the height. This agrees with the polarization feature and the vectoral nature of the aperture radiation. As shown in Fig. 3 in the main text, the radiation through the aperture is much broader in the vertical direction than in the horizontal direction, because the beam is mainly polarized in the vertical direction and such radiation should be described by the vector Stratton-Chu model instead of the scalar optical theories. It is also consistent with our discussion in Supplemental Material Sec. A, especially Fig. S3 that the focusing effect of the metallic aperture is related to the change in the boundary conditions near the left and right poles of it.

C Additional information on the focused ion beam milling

Figure S9 shows the workflow of patterning the metallic coating with FIB milling, and corresponding images at different steps are shown on the right side. These images are taken for the same device as characterized in Figs. 2, 3 in the main text.

Start from a mounted device. At least two sets of markers are milled by FIB on the semiconductor facets around 20 μm away from the active region waveguide for relocating the waveguide after coating deposition. Metallic coatings consisting a sequence of Al_2O_3 and Au layers are deposited via electron beam evaporation on both the front and the back facets of the laser. The deposition rate needs to be calibrated in order to control the thicknesses, especially for the dielectric layer. Circular patterns are milled through the metallic film with FIB milling. After that, the alignment between the aperture and the waveguide can be checked with the energy-dispersive X-ray (EDX) spectroscopy as shown in the bottom right image of Fig. S9. In order to penetrate the thick coating layers, high operating voltages and currents are needed.

While performing the FIB milling, we also take real-time SEM and FIB images to monitor the milling progress. In this way, we are able to stop the milling immediately as soon the Au layer is fully patterned through. The milling rate of Al_2O_3 is much smaller than the one of Au given the same conditions. In experiments, the Au layer thickness is around or less than 200 nm. A circular aperture with 1 μm diameter through such a film requires only few seconds at a FIB operating current of 26 pA and the voltage of 30 kV. Because we are monitoring the milling process in real time and the milling rate difference between Al_2O_3 and Au is huge, the milling into the Al_2O_3 layer is negligible, if there is any.

Figures S10(a,b) show the side-view photos of the vacuum chamber when conducting the FIB milling on the front (a) and back (b) facet coatings. The electron beam gun is in the vertical direction (90°) and the ion beam gun is at the oblique direction ($\approx 52°$). The operating facet of the sample should be rotated to the direction normal to the ion beam gun for the FIB milling.

Performing the FIB milling on the back facet is essentially the same as doing it on the front facet, both of which are described by the workflow shown in Fig. S9. The only difference is that when operating the back facet milling, the device is placed on a customized 45° holder and the orientation of the device is flipped such that the back facet is facing the ion beam gun, as shown in Fig. S10(b).

D Additional measurement results of the threshold reducing experiment

In addition to the current-voltage-power characterization of the presented laser (Device I) at 20 °C, which is shown as Fig. 2 of the main text, measurements with a current step of 0.1 mA are conducted also at 0 °C and -20 °C, as shown in the right insets Fig. S11(a). The threshold currents are 9.6 mA and 8.3 mA at 0 °C and -20 °C, respectively.

The patterned coating technique is also implemented on the front coatings of three other devices (Devices II, III, IV) with the same material, similar dimensions and similar coating structures as Device I. Clear improvement in the threshold currents (around or larger than 1 mA) is observed in all these devices at all measured temperatures, proving that presented threshold-reducing technique is reliably reproducible. The structural details of the four devices are listed in Tab. S1.

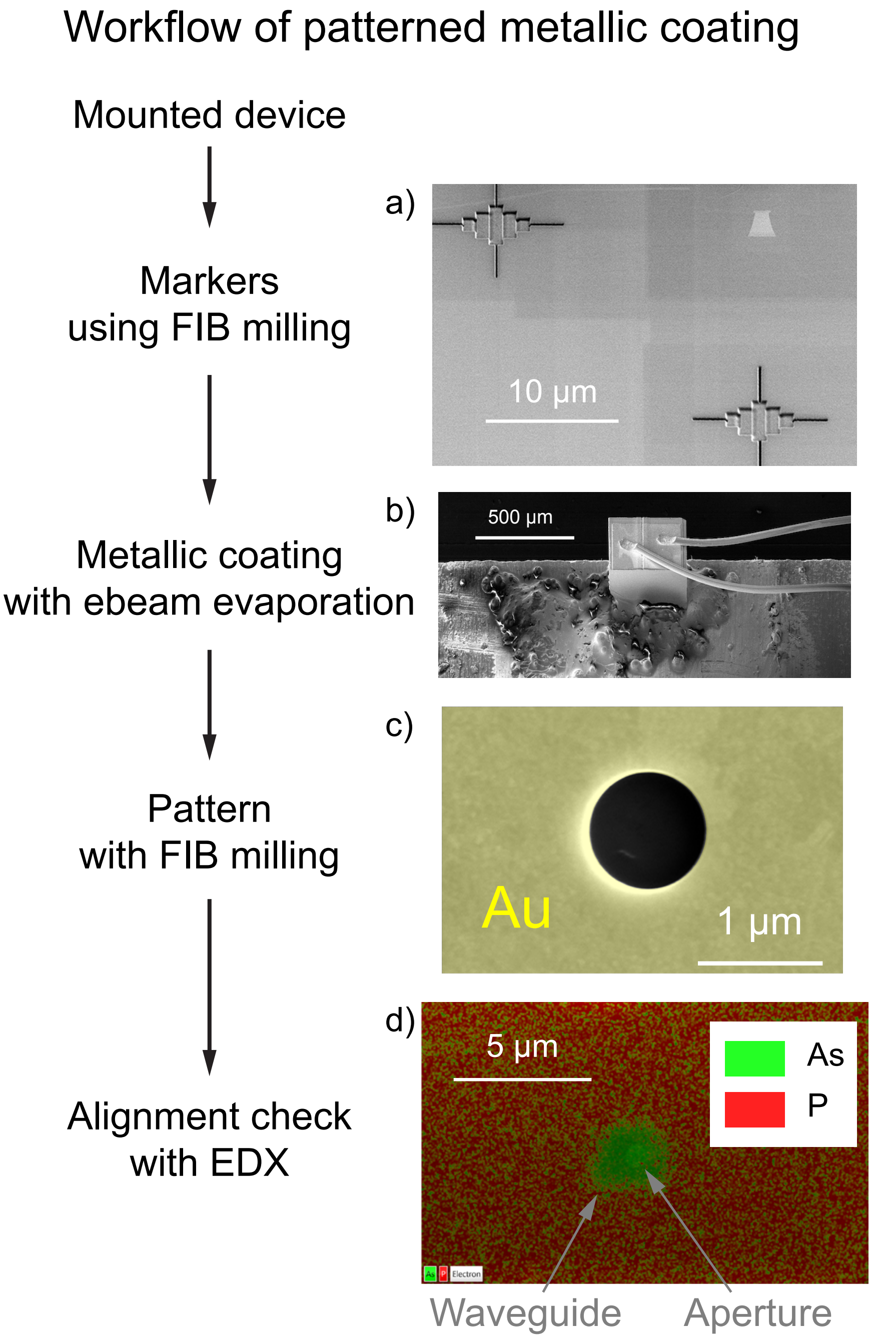

Figure S9. Aperture milling workflow. Workflow of the patterned metallic coating on a processed laser. Corresponding SEM images at different steps are shown on the right side. The Au film is manually colored as yellow in (c). FIB milling: focused ion beam milling. EDX: energy-dispersive X-ray.

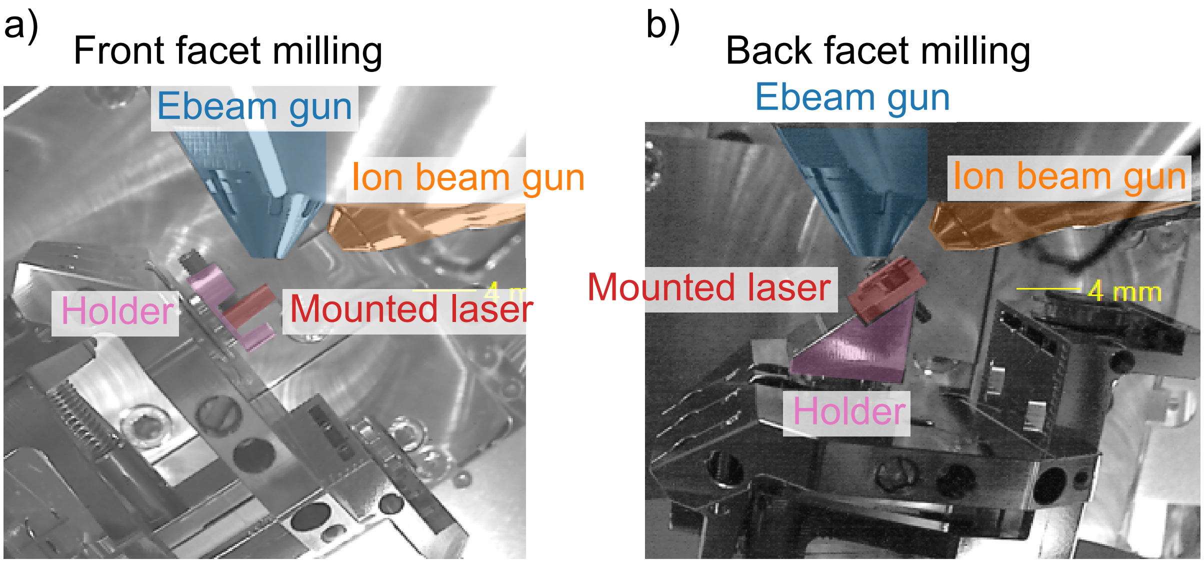

Figure S10. Photos of the FIB chamber. Side-view images of the vacuum chamber when conducting the FIB milling on the front (a) and back (b) facets of the coated lasers. The images are colored manually.

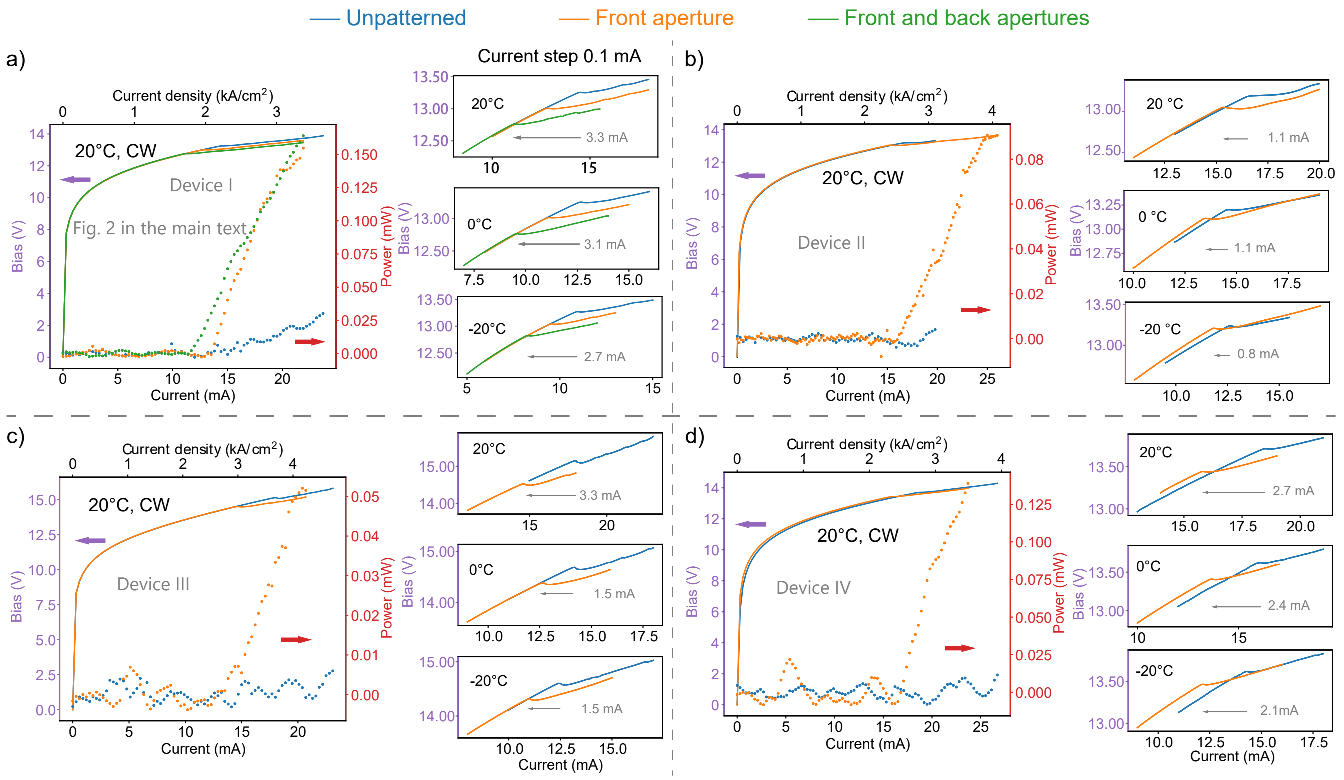

Figure S11. Reproducibility of the threshold reduction. Continuous-wave (CW) current-voltage-power characteristics of four devices at room temperature and detailed current-voltage measurements (current step 0.1 mA) at 20 °C, 0 °C and -20 °C. Device I in (a) is the same device as presented in the main text.

ID	Aperture diameter (nm)	Al_2O_3 thickness (nm)	Au thickness (nm)	Avg. waveguide width[i] (μm)	Min. waveguide width[ii] (μm)	Device length (μm)	Mounting
Device I	950	700	200	2.8	2.5	265	Epi-up
Device II	850	692	120	3.2	2.6	250	Epi-up
Device III	960	786	200	2.1	1.6	305	Epi-down
Device IV	950	786	200	2.9	2.6	260	Epi-down

Table S1. Structural details of the four devices presented in Fig. S11.
[i] Average waveguide width, also used for simulating the optical field in COMSOL 3D, where the waveguide is approximated as a rectangular shape.
[ii] Minimum waveguide width: width of the narrowest part of the waveguide, used for calculating the current density.

E Additional details of the far-field and near-field patterns

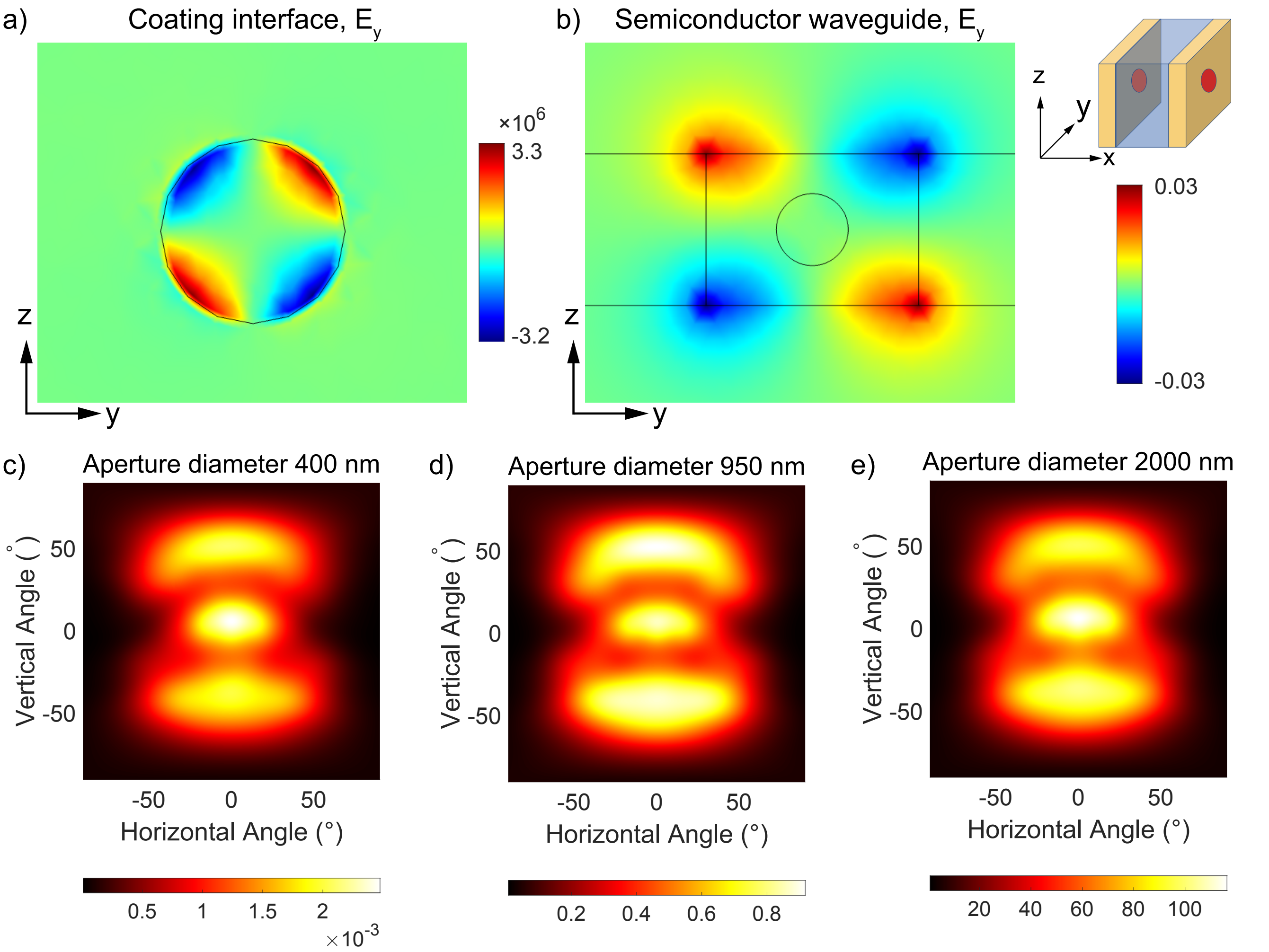

Figure S12. Near-field and far-field patterns. (a,b): Simulated near-field patterns of the horizontal electric field component ($\mathbf{E}_y$) at: (a) the interface between the high-reflectivity coating and the free space; (b) cross-section of the semiconductor waveguide. The upper right inset indicates the definition of the coordinate axis. (c-e): Simulated far-field patterns of the beam emitted through the metallic aperture for three different aperture sizes. (d) is the same plot as Fig. 3(g) in the main text.

As shown by Fig. 3(i) in the main text, the horizontally polarized far-field pattern is anti-symmetrical and is dark at

the center [note that Figs. 3(a-i) are showing the patterns of the far-field power $|\mathbf{E}|^2$]. As shown in Fig. S12(a,b), this anti-symmetrical property is consistent with the near-field patterns of the horizontal electric field (E_y) at the aperture interface, which is determined by the symmetry of the incident waveguide mode. Fundamentally, the horizontal field components arise from the field continuity conditions at the edges of the waveguide.

The unpolarized far-field patterns of the light emitted through differently sized apertures are simulated and shown in Figs. S12(c-e). Being simulated with identical parameters, Fig. S12(d) is in fact the same plot as Fig. 3(g) in the main text. By comparing Figs. S12(c-e), it is shown that although the near-field emitting dimension is enlarged by a factor of 5 (25 times in the area), the change in far-field beam divergence is almost invisible, especially along the vertical direction. This indicates that the far-field must be calculated by taking into account the vector nature of the optical field and the classic scalar approximation is no longer valid, as mentioned in the main text.

For the uncoated laser facet, COMSOL simulation shows that the peak power ratio between the horizontally polarized far-field beam and the vertically polarized one is 4%. In the case of the metallic coating with a 950 nm-diameter aperture as presented, this ratio is increased to 24%, showing that the relative intensity of the far field in the horizontal polarization is significantly enhanced by the presence of the metallic aperture, as mentioned in the main text.

F Reflectivity enhancement in TE modes at the wavelength of 1550 nm

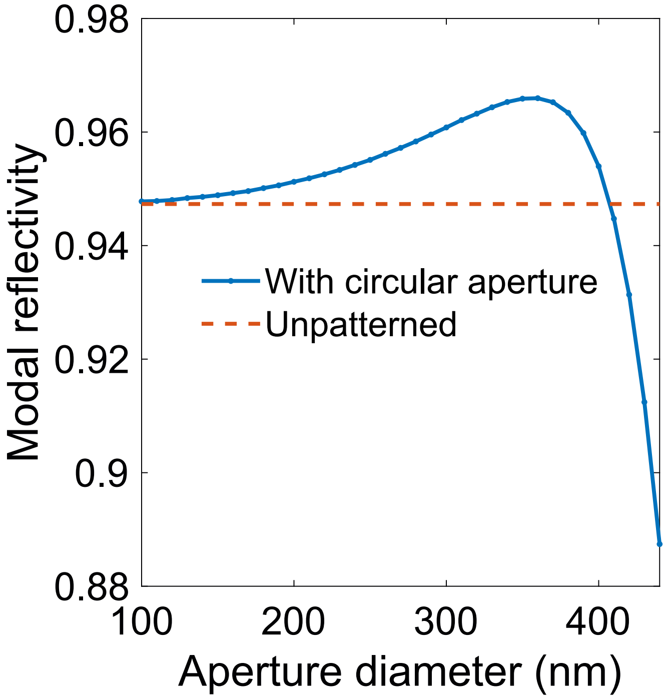

Figure S13. Reflectivity enhancement at 1550 nm wavelength. Modal reflectivity of the metallic coating as a function of the circular aperture diameter, for TE polarized mode at the wavelength of 1550 nm. The insulating layer thickness is set as 300 nm. The red dashed line shows the reflectivity of the coating without an aperture.

The presented effect of the reflectivity enhancement is not limited to QCL devices. In the main text, the demonstration is conducted with transverse magnetic (TM) polarized modes at the wavelength of 4.5 μm. Figure S13 shows the modal reflectivity dependence on the aperture diameter of a similarly patterned metallic coating at the wavelength of 1550 nm, plotted in the same way as Fig. 1(c) in the main text. Here the laser structure is composed of rectangular shaped waveguide surrounded by low index InP material. The waveguide mode is transverse electric (TE) polarized. The waveguide has a width of 800 nm, a height of 500 nm and a refractive index of 3.30. The refractive index of the surrounding InP is set as 3.16. The metallic coating

consists of 300 nm Al_2O_3 layer and 100 nm Au layer. The aperture is aligned to the center of the waveguide.

It is clearly shown in Fig. S13 that the modal reflectivity is enhanced by introducing the circular metallic aperture into the Au film. The modal reflectivity is maximized at a diameter of 360 nm and the mirror loss is reduced by 35% compared to the case without any aperture. This result confirms that the reflectivity enhancement by a subwavelength metallic aperture is not exclusive to TM-polarized QCL devices.

G Transmission enhancement at a large aperture dimension

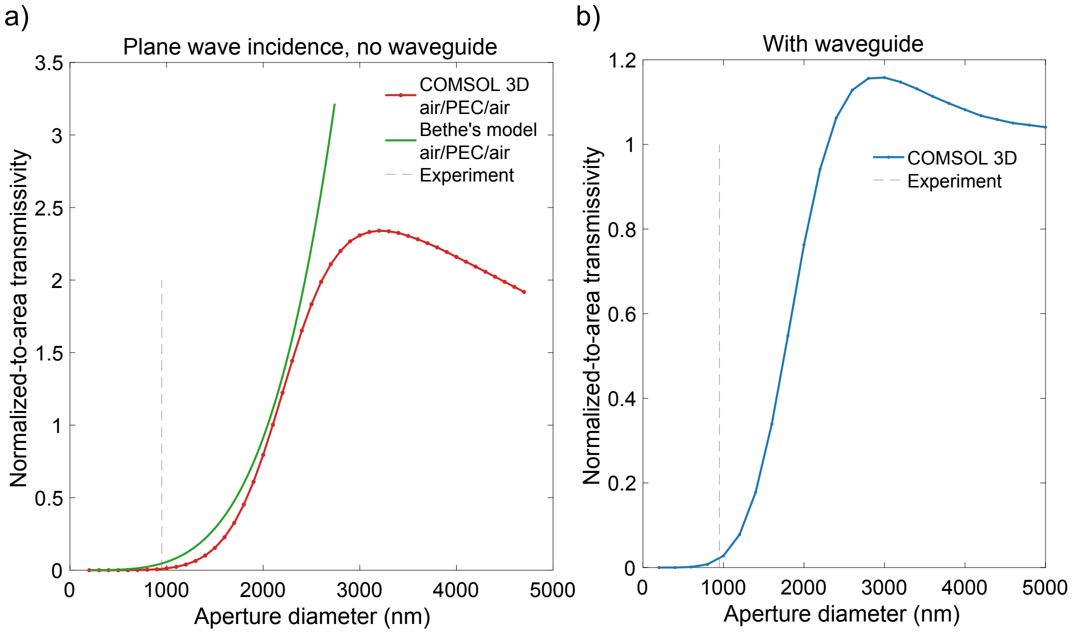

Figure S14. Transmission enhancement through the metallic aperture. (a) Normalized-to-area transmittance as a function of the aperture diameter for a normally incident plane wave onto a perforated PEC film. The red dotted line shows the results obtained by COMSOL 3D simulation with a 200 nm thick film placed in air. The green solid line shows the results calculated with Bethe's model. The gray dashed line indicates the aperture size in the reflection-enhancement experiments of this manuscript. (b) Normalized-to-area transmittance as a function of the aperture diameter for the waveguide HR coating perforated by a circular aperture. The normalized-to-area transmittance is calculated by having the transmitted power divided by the incident power within the aperture area. The dielectric layer thickness is 700 nm.

Although it is not the primary goal of this work, transmission enhancement is observed in 3D COMSOL simulations when the aperture dimension is far from the reflectivity enhancement domain. The simulations are performed for two cases, plane-wave incidence and diverging guided mode incidence. In the first simulation, a linearly polarized plane wave with the wavelength of 4.5 μm is normally incident on a 200 nm-thick metal film which is ideally approximated as a perfect electric conductor (PEC). A circular aperture is opened through the metallic film and the normalized-to-area transmittance of the aperture is shown in Fig. S14(a). The PEC film is placed in air. In the small radius limit, the results simulated by COMSOL 3D (red dotted line) agrees with Bethe's model (green line)[1].

Figure S14(b) shows the simulated results of the second case, i.e. the perforated metallic coating of the laser waveguide where the aperture is incident by a diverging beam. The Al_2O_3 layer thickness is 700 nm. The maximum normalized-to-area transmission is 1.16 when the aperture diameter is near 3000 nm. Although this value is above unity, the enhancement effect is limited and we here clarify that this is not extraordinary optical transmission.

The parameter conditions for the above-unity normalized-to-area transmissivity are far away from the parameters we used in the the experiments of this work (Fig. 2 in the main text), as indicated by the gray dashed lines where $2r = 950$ nm. We again emphasize that this transmission enhancement does not align with the major scope of interest in this work.

References

1. Bethe, H. A. Theory of diffraction by small holes. Phys. Rev. **66**, 163 (1944).
